# Disparities of Mental Health Service Needs and Utilization Among Older Adults

**DOI:** 10.1093/geroni/igab046.1584

**Published:** 2021-12-17

**Authors:** Yuanyuan Hu, Qingwen Xu

**Affiliations:** 1 New York University, Elmhurst, New York, United States; 2 New York University, New York University, New York, United States

## Abstract

Prior studies have examined mental health disparities, however, without adequate attention to the older adult population. Framed by the Andersen Behavioral Model of Health Service Use, this study was to examine the prevalence of depression and anxiety and the mental health service use among older adults of different race/ethnicity; and to investigate factors associated with mental health services use(counseling and psychotropic medication). Data from the National Health Interview Survey 2019 were analyzed by bivariate tests and logistic regression analyses. Hispanic older adults have the highest rates of depressive and anxious symptoms, followed by Whites, Blacks, and Asians. Non-Hispanic Asians and Blacks reported significantly lower rates of taking medication. The severity of depression and anxiety was consistently associated with mental health service use across all groups. Education was positively associated with counseling use in white and black groups. For older whites, better general health, male and foreign-born were significantly predicting less medication use. Older blacks with better general health were significantly less likely to use medication. For Hispanic older adults, female and being single were associated with anxiety medication use. Results suggest that older adults, despite different perceptions and cultural understandings of mental health, use mental health services for severe conditions. This study also highlights the important role that education and health literacy could have played in the use of counseling services. For the medication use, the result—that general health status was important for both black and white older adults, but not Hispanics—could suggest a few directions for further exploration.

